# Brain2Pix: Fully convolutional naturalistic video frame reconstruction from brain activity

**DOI:** 10.3389/fnins.2022.940972

**Published:** 2022-11-14

**Authors:** Lynn Le, Luca Ambrogioni, Katja Seeliger, Yağmur Güçlütürk, Marcel van Gerven, Umut Güçlü

**Affiliations:** ^1^Donders Institute for Brain, Cognition and Behaviour, Radboud University, Nijmegen, Netherlands; ^2^Max Planck Institute for Human Cognitive and Brain Sciences, Leipzig, Germany

**Keywords:** visual reconstruction, decoding, neural networks, vision, fMRI

## Abstract

Reconstructing complex and dynamic visual perception from brain activity remains a major challenge in machine learning applications to neuroscience. Here, we present a new method for reconstructing naturalistic images and videos from very large single-participant functional magnetic resonance imaging data that leverages the recent success of image-to-image transformation networks. This is achieved by exploiting spatial information obtained from retinotopic mappings across the visual system. More specifically, we first determine what position each voxel in a particular region of interest would represent in the visual field based on its corresponding receptive field location. Then, the 2D image representation of the brain activity on the visual field is passed to a fully convolutional image-to-image network trained to recover the original stimuli using VGG feature loss with an adversarial regularizer. In our experiments, we show that our method offers a significant improvement over existing video reconstruction techniques.

## 1. Introduction

A long-lasting interest of sensory neuroscience is understanding how sensory information is represented in neural activity patterns. Decoding visual stimuli from neural activity using deep learning is a promising approach for bringing us closer to such understanding. Recent advances allow the successful decoding of static images from brain data (Thirion et al., 2006; Miyawaki et al., 2008; Naselaris et al., 2009; van Gerven et al., 2010; Kok et al., 2012; Horikawa et al., 2013; Güçlütürk et al., 2017; Seeliger et al., 2018; Dado et al., 2020). Reconstructing natural movies is significantly more challenging (Nishimoto et al., 2011) yet important given that neurons respond to signals that unfold over both space and time (Nishimoto and Gallant, 2011). The difficulty with reconstructing natural movies is in large part due to the limited temporal information provided by imaging methods such as fMRI as well as the complex dynamics of the natural world that the model must learn.

Convolutional image-to-image models have recently achieved unprecedented results in multiple tasks such as semantic segmentation (Long et al., 2015; Noh et al., 2015; Ronneberger et al., 2015; Li et al., 2017; Zhang et al., 2018a), style transfer (Güçlütürk et al., 2016; Selim et al., 2016; Isola et al., 2017; Zhu et al., 2017), colorization (Iizuka et al., 2016; Zhang et al., 2016, 2017), and super-resolution (Dong et al., 2016; Kim et al., 2016; Zhang et al., 2018b). Convolutional image-to-image networks have the great advantage of preserving the topography of input images throughout all the layers of the network. Consequently, the network does not need to learn a remapping between locations and can focus on processing local features. The reconstruction of perceived natural images from brain responses can be considered as a form of image-to-image problem since the visual cortex processes information in a topographically organized manner (Henschen, 1893; Inouye, 1909; Holmes and Lister, 1916) such that the topology of the input images is preserved within each visual area. The retinotopic mapping of visual neurons defines relationships between the visual field and its cortical representation in individual subjects and has uncovered many important aspects of the visual cortex across different species (Hubel and Wiesel, 1962; Dumoulin and Wandell, 2008). However, it is not straightforward to exploit this in an image-to-image ConvNet architecture. The cortex itself can be roughly seen as a pair of topological spheres embedded in a 3D space. Several separate visual representations are embedded in this cortical space, corresponding to several visual areas (e.g., V1, V2, V3). These representations are furthermore distorted by the geometry of the cortex and by the uneven sampling of different parts of the visual field. Therefore, there is no natural way of constructing a convolutional architecture that exploits the image-to-image nature of the problem by preserving the topography between voxel responses and pixel brightness and color.

In this paper, we exploit the receptive field mapping of visual areas to convert voxel responses defined in the brain to activations in pixel-space. Early visual areas V1, V2, and V3 were identified using retinotopy. The voxel activations of each area are then converted to images via the mapping of the receptive fields. Importantly, these images (visual representations) do have a pixel-to-pixel correspondence with the images used as stimuli. We then transform these visual representations into realistic images using an image-to-image U-network trained using a combination of pixelwise, feature, and adversarial losses.

## 2. Related work

Recent work on image reconstruction from fMRI data has demonstrated the success of employing deep neural networks (DNNs) and generative adversarial networks (GANs) in neural decoding (Nishimoto et al., 2011; Güçlütürk et al., 2017; Horikawa and Kamitani, 2017b; Wen et al., 2017; Seeliger et al., 2018; Han et al., 2019; Shen et al., 2019a,b). For instance, Seeliger et al. (2018) used a GAN to reconstruct grayscale natural images as well as simpler handwritten characters. Another approach was voxel-wise modeling by Nishimoto et al. (2011), where they modeled responses to complex natural stimuli to estimate the semantic selectivity of voxels. More recently, Shen et al. (2019a) showed that even with a limited set of data—in the order of thousands compared to millions that the reconstruction field is accustomed to—it was possible to train an end-to-end model for natural image stimulus reconstruction by training a GAN with an additional high-level feature loss. Their reconstructions matched several high-level and low-level features of the presented stimuli. However, a comparable performance has not yet been achieved for naturalistic video stimuli. The most recent notable video reconstruction study by Han et al. (2019) made use of a variational auto-encoder and was able to reconstruct low-level properties of the images, where the reconstructions resembled shadows or silhouettes of the stimulus images. Reconstruction of perceived videos can thus be considered a very challenging problem.

The simplest way to apply convolutional neural networks (ConvNets) on fMRI voxel responses is to treat fMRI slices as separate images stacked on the channel dimension (Sarraf and Tofighi, 2016). However, these images do not respect the topography of neural representations and contain a large fraction of non-responsive voxels corresponding to white matter and cerebrospinal fluid. This results in most of the contrast of the images depending on irrelevant anatomical factors. Another possibility is to use spatial 3D convolutions on the brain volume (Bäckström et al., 2018). This method has the benefit of preserving the topography of the neural responses but otherwise has the same issues as the 2D approach. These shortcomings make such methods unsuitable for brain decoding and reconstruction. A more viable strategy is to map the voxel responses on a mesh representing the cortical surface (Fischl, 2012) and apply a geometric deep learning technique (Monti et al., 2017; Cohen et al., 2018; Fey et al., 2018; Kondor et al., 2018).

## 3. Materials and methods

Our brain2pix architecture has two components: (1) a receptive field mapping that transforms the brain activity of visual regions to a tensor in pixel (input) space; (2) a pix2pix network that converts the brain responses in pixel space to realistically looking natural images. In the following, we describe the two components in detail.

### 3.1. From voxels to pixels

A receptive field mapping is a (potentially many-to-one) function that maps the 3D coordinate of the voxels of a visual area to Cartesian coordinates in the stimulus space. This coordinate is defined as the region of the image that elicits the highest response in the voxel. Given a visual ROI (region of interest), we can refer to these mappings using the following notation:


(1) $$\begin{array}{l} \text{RF}\left(r_{1},r_{2},r_{3}\right)=\left(x,y\right)\;, \end{array}$$

where (*r*_1_, *r*_2_, *r*_3_) are the voxel coordinates and (*x, y*) are Cartesian coordinates in stimulus/pixel space in the image space. Since visual areas are topographically organized, this map can be seen as an approximate homeomorphism (i.e., a function that preserves the topology). Note that RF does not respect the metric structure of the image since the representation of the fovea is inflated while the periphery is contracted. We denote the function associating a measured neural activation (BOLD response) to each voxel as *n*(*r*_1_, *r*_2_, *r*_3_). Using the receptive field mapping, we can transport this activation map to pixel space as follows:


(2) $$\begin{array}{l} n\left(x^{\prime },y^{\prime }\right)=\frac{1}{M\left(x^{\prime },y^{\prime }\right)}\underset{\begin{array}{c} r_{1},r_{2},r_{3};\text{RF}\left(r_{1},r_{2},r_{3}\right)=\left(x^{\prime },y^{\prime }\right) \end{array}}{\sum }n\left(r_{1},r_{2},r_{3}\right)\;, \end{array}$$

where *M*(*x*′, *y*′) is the number of voxels that map to the coordinates (*x*′, *y*′). Equation (1) is limited to the case of point-like receptive fields. More generally, the RF transport map can be written as a linear operator:


(3) $$\begin{array}{l} n\left(x^{\prime },y^{\prime }\right)=\underset{r_{1},r_{2},r_{3}}{\sum }W_{r_{1},r_{2},r_{3}}^{x^{\prime },y^{\prime }}n\left(r_{1},r_{2},r_{3}\right)\;, \end{array}$$

where the weight tensor *W* is a (pseudo-)inverse of the linear response function of the cortex under single pixel simulations. This second formulation has the benefit of allowing each voxel to contribute to multiple pixels and to be suitable to gradient descent training.

In this paper, we use two strategies for determining *W*. The first approach, is to apply an off-the-shelf receptive field estimator and to use Equation (1). The second, more machine learning oriented approach, is to learn a weight matrix together with the network. In order to preserve the topographical organization, we include the learnable part as a perturbation of the receptive field estimation:


(4) $$\begin{array}{l} n\left(x^{\prime },y^{\prime }\right)=\underset{r_{1},r_{2},r_{3}}{\sum }\left(\frac{\delta_{\text{RF}\left(r_{1},r_{2},r_{3}\right)}^{\left(x^{\prime },y^{\prime }\right)}}{M\left(x^{\prime },y^{\prime }\right)}\cdot V_{r_{1},r_{2},r_{3}}^{x^{\prime },y^{\prime }}\right)n\left(r_{1},r_{2},r_{3}\right)\;, \end{array}$$

where $\delta_{x}^{y}$ is the discrete delta function which represents a boolean mask to determine whether at each coordinate there is a response or not. If there is a response, it is 1 and if there is no response it becomes a 0. The weights $V_{r_{1},r_{2},r_{3}}^{x^{\prime },y^{\prime }}$ are learnable parameters.

### 3.2. Image-to-image network

The input to the pix2pix network is a tensor obtained by stacking the voxel activation maps, one map for each combination of ROI and time lag. In fact, the network needs to integrate the topographically organized information contained in several layers of the visual hierarchy (V1, V2, and V3 in our case) but also the responses at different time lags (the network selects from the five provided inputs).

#### 3.2.1. Architecture

**Figure 1:**
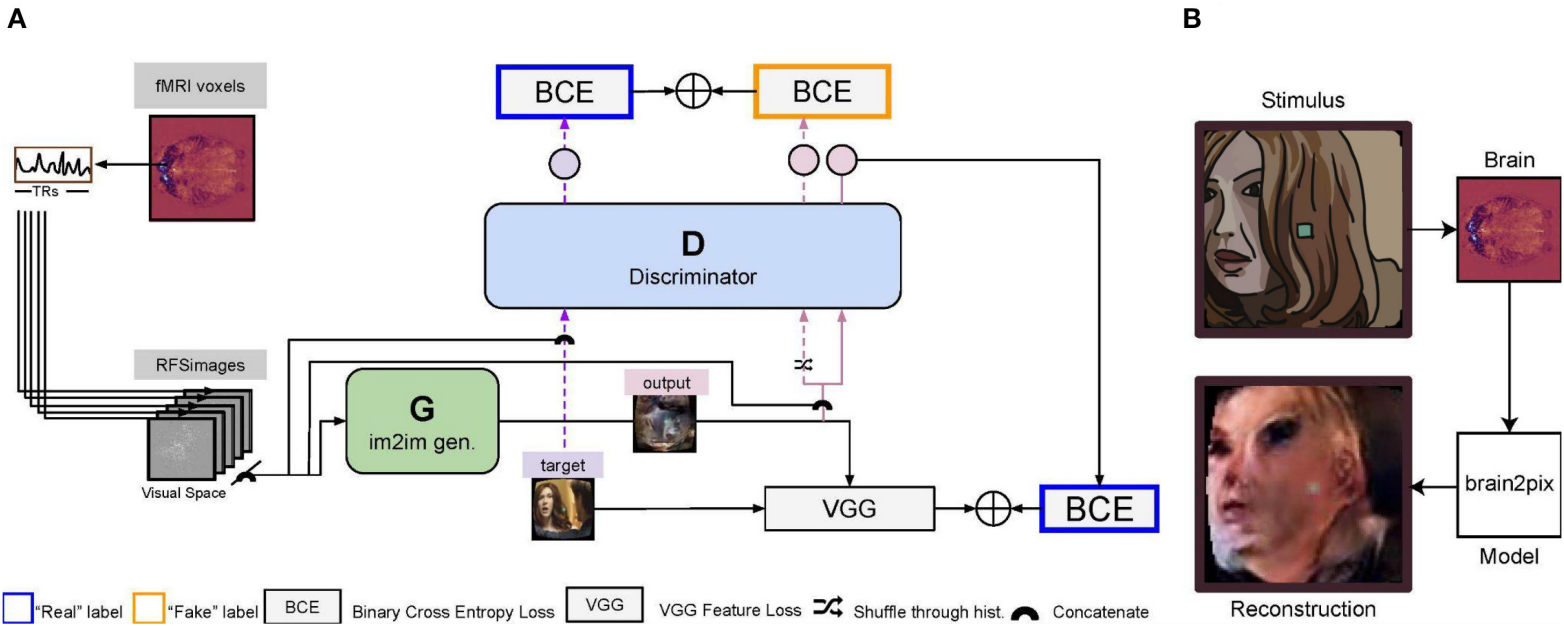
Illustration of the brain2pix model. **(A)** First, the voxels of each region of interest (ROI) voxels are extracted. The voxel receptive fields are then projected onto the input (visual) space. Using this mapping, the 2D receptive field signal images (RFSimages) are created, which reflect voxel activity at the locations of their receptive fields in the visual space. To account for the hemodynamic delay, these activities are taken with a fixed delay of 5 TR (i.e., the activities are taken from the volume recorded approximately 4 s after stimulus presentation). The input to the **generator** are the RFSimages, and the outputs are the reconstruction (end-to-end modeling). The loss is estimated between the target (original) image and this reconstruction as a feature loss (VGG loss). The reconstruction is also passed through the discriminator, concatenated with the RFSimages. The loss for training the **discriminator** is obtained by summing its output based on the reconstructed image and the target image (concatenated with the RFSimages), using a binary cross entropy (BCE) loss. The BCE loss estimated on the discriminator's output is summed with the VGG loss, and this sum of losses is then used to backpropagate and update the parameters of the generator. **(B)** Example reconstruction on the test set. The end-to-end model reconstructs static video frames, using the brain signal of the participant watching Doctor Who in the fMRI scanner as the input.

The Pix2Pix architecture (Isola et al., 2017) comprises a convolutional U-Net-based generator (Ronneberger et al., 2015) and a convolutional PatchGAN-based discriminator. The first and the last layers of the generator are, respectively, convolutional and deconvolutional with four standard U-net skip blocks in-between. All five layers of the discriminator are convolutional with batch normalization and a leaky ReLU activation function. Together with an L1 loss, this model translates images to images. Our Brain2Pix model is inspired by this concept and also makes use of a discriminator and generator (which we call the adversarial loss). We combine the adversarial loss with an L1 loss and a feature loss, to translate brain signals mapped onto 2D space (RFSimages) to reconstructions. Our feature is implemented by extracting features of the targets and the generator's output from a pre-trained VGG loss. The differences between these features are summed with the adversarial loss and L1 loss to update the parameters of the generator (Figure 1).

The discriminator was trained to distinguish stimuli from their reconstructions by iteratively minimizing a loss function with a sole adversarial loss [binary cross-entropy (BCE)] and using a history buffer to encourage the discriminator to remember past errors. The history buffer has two functionalities; to save reconstructions from the generator and to be sampled from. Before any forward passes, an empty history buffer is made with a capacity set to 50 reconstructions. As the batches are passed through the network for training, the buffer is filled with up to 50 reconstructions from the generator. Once the capacity of the history buffer has been reached, there is 50% that the reconstructions going into the discriminator is swapped with a randomly selected reconstruction from the history buffer during training. This way, there is always a chance that the discriminator makes use of a reconstruction from the past to learn, rather than only using the output of the most up-to-date generator.

The generator was trained for converting brain responses to stimulus reconstructions by iteratively minimizing a loss function with three weighted components: (i) pixel-loss, which was taken to be the absolute difference between ground-truths and predictions, (ii) feature loss, which was taken to be the Euclidean distance between pre-trained layer 10 VGG features of ground-truths and predictions, and (iii) adversarial loss, which was taken to be the “inverse” of the adversarial loss that was used to train the discriminator. The implementation of the loss is visualized in Figure 1.

#### 3.2.2. Training configurations

Each loss component has a weight, which determines how much of an affect each loss would have on the final results. Both the adversarial loss and the L1 loss had a weight of 1, the feature loss had a weight of 100. Optimization was done with the Adam optimizer with a beta1 of 0.5 and a learning rate of 0.0002.

All models were implemented in Python with the MXNet framework (Chen et al., 2015). They were trained and tested on Nvidia GeForce 2080 Ti GPUs.

#### 3.2.3. Receptive field estimation

Receptive fields for dorsal and ventral visual regions V1, V2, and V3 were estimated in a data-driven way using neural information flow (Seeliger et al., 2021). Grayscale video sections were passed through three 3D convolutional neural network layers corresponding to the visual ROIs. Before the ROI-specific layers a linear layer with a single 1 × 3 × 3 channel was used to allow learning retinal and LGN preprocessing steps. Average pooling was applied after each layer to account for increasing receptive field sizes, the temporal dimension was average pooled to a TR of 700 ms before applying the observation models, and spatio-temporal receptive fields were constrained to be positive. For training this neural network, a low-rank tensor decomposition was applied to estimate voxel-wise spatial, temporal, and channel observation (readout) vectors, which were used to predict voxel-wise activity from the neural network activity tensors. The receptive field location (*x, y*) for every voxel was then estimated as its center of mass of the low-rank receptive field maps.

### 3.3. Data acquisition

We made use of a public large fMRI dataset from single-participant responses to naturalistic video stimuli (Seeliger et al., 2019). The exact experiments are described in detail in the original study (Seeliger et al., 2019). In short, the participant fixated on the center of the screen and watched 30 episodes of BBC's Doctor Who while their BOLD activity was measured. The videos were presented in multiple sessions, using a head cast for positional stability and consistency. The recording comprised presenting 30 full episodes once (forming the training set, here used for model estimation), and seven short clips—teasers and short stories, with a Doctor played by a different actor to avoid train-test overlap—repeatedly shown at the end of most sessions (forming the test set). The 22–26 repetitions of the test set were averaged and used for evaluation of the brain2pix model. The averaged test set is common in neural coding and most reconstruction research to provide a version of the individual's BOLD response patterns with high signal-to-noise ratio, freed of the substantial noise contained in fMRI data. To assess the implications of reconstruction research it is important to understand that the presented reconstructions have so far usually been estimated on data from specific individuals averaged over multiple presentations in highly controlled settings.

Data collection was approved by the local ethical review board (CMO regio Arnhem-Nijmegen, The Netherlands, CMO code 2014-288 with amendment NL45659.091.14) and was carried out in accordance with the approved guidelines. For every session written formal consent was obtained from the participant.

### 3.4. Data preprocessing

Prior to using the inputs for training the model, 3D brain matrices were transformed to 2D receptive field signal images (RFSimages) in two main steps. First, regions of interests (ROIs) were selected from the brain (V1, V2, V3), based on their corresponding masks. Second, voxels in each brain region were mapped onto their corresponding visual space based on the retinotopic map. More specifically, the brain signals are shifted and stacked with 5 TRs per corresponding stimuli. This resulted in an array of (f, TR, s, 1, 1) dimensions per brain region. With f being the amount of frames in the dataset, TR being the brain signals of five consecutive time-points, s being the amount of voxels in that particular ROI, and the remaining dimensions are left empty to later multiply with a 96 by 96 RF map to form the 2D RFSimages.

The videos were downsampled spatially (96 × 96 × 3) and temporally to match the TRs of the fMRI recordings (one frame every 0.7 s). We did this by using the ffmpeg software by running

ffmpeg -i original-video.webm

-an -vf ~scale=96:96,fps=1.42857142857~

-y output-video.webm

for every run in the training and testing dataset. Then all the webm files are converted to numpy arrays using the openCV library and concatenated to become a file per run.

This resulted in a total of approximately 119.000 video frames for training and 1,034 video frames for model evaluation. To incorporate the hemodynamic delay we realigned the stimuli and brain signals such that the current signals correspond to the stimuli that were presented at 5 TRs before, allowing a time window of 2.1–4.2 s delay from stimulus presentation (since we are incorporating 5 TRs). Finally, each frame underwent a fish-eye transformation, which mimics biological retinal eccentricity (Bashivan et al., 2019). The receptive field centers we used for mapping brain signals onto the visual space were based on images that underwent this transformation.

### 3.5. Experimental design

We compared our final model with alternative reconstruction models. This included a baseline comparison where brain2pix was compared with previously suggested models. We wanted to focus on early visual areas, so we trained our model on V1, V2, and V3 individually (which we called the ROI experiment) using the retinotopic maps provided by the paper that provided the dataset (Seeliger et al., 2019). Finally, we tested whether our model was robust to various ablations.

**Figure 2:**
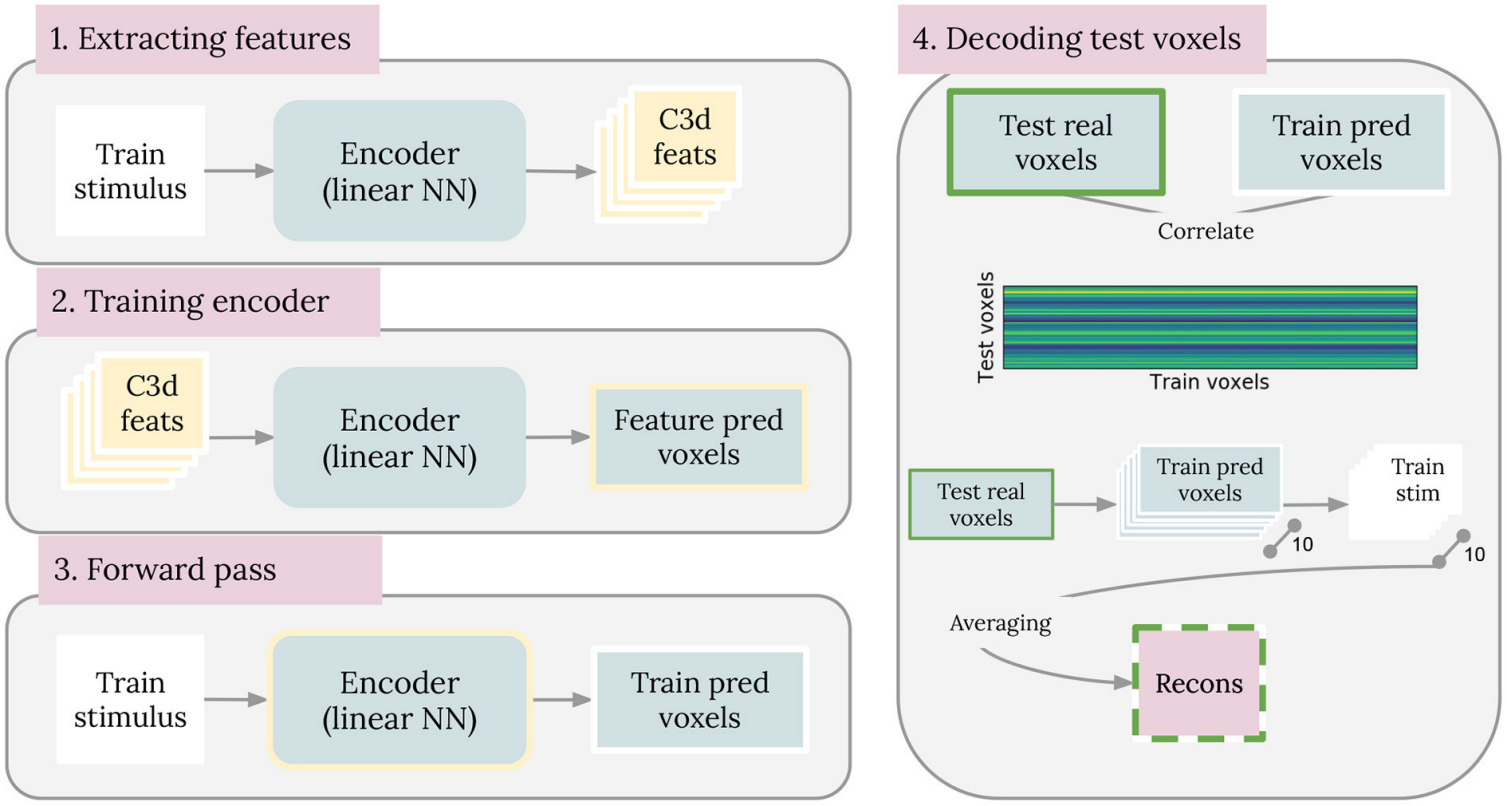
Baseline 1 method. Steps that were taken to implement the Nishimoto-like model as baseline 1 are illustrated here. Steps are described in full details in section 3.5.

We compared our brain2pix results with baseline models based on state-of-the-art reconstruction models. The first baseline is similar to the method introduced by Nishimoto et al., which used a set of naturalistic videos as an empirical prior (Naselaris et al., 2009; Nishimoto et al., 2011). In our experiment, we used a smaller but more targeted natural image prior constructed from the training set. We trained an encoding model, consisting of a dense layer, that predicts BOLD activity from C3D features extracted from training samples, which we used as the likelihood. We do so by first extracting c3d features are from every image in the training set (1). Then, the c3d features are used as input to train an encoding model, with its corresponding voxel as output (Feature pred voxels) (2). Once the encoder is trained, a forward pass is performed using all the stimuli from the training dataset (Train stimulus) and the predicted voxels are saved (Train pred voxels) (3). Finally, a correlation matrix is made between the voxels from the testing dataset (Test real voxels) and the Train pred voxels (4). Then, we constructed the images by averaging the 10 clips with the highest likelihood. The steps are shown in Figure 2.

The second baseline model we trained with an adversarial loss and a feature loss, which is based on the end-to-end reconstruction model from Shen et al. (2019a). We used the generator and discriminator modules present in brain2pix, however, we did not construct RFSimages for the input of the model. Instead, this baseline model takes as input the fMRI voxels that are z-scored and temporally aligned with the stimuli (same pre-processing steps as the original brain2pix), and the first layer is a linear layer as it does not take in voxels that are mapped onto 2D space.

The same four evaluation metrics were used in all experiments, namely Pearson's product-moment correlation coefficient (corr.) and Euclidean distance (dist.) between the features of the presented test stimuli and their reconstructions. Features were extracted from the pool2, pool5, and fc6 layers of the AlexNet model (Krizhevsky et al., 2012) and the C3D model (Tran et al., 2015). The Alexnet model was pre-trained on ImageNet (Russakovsky et al., 2015) and the C3D model was pre-trained on Kinetics-400 (Kay et al., 2017).

The novelty of the current work is the application of existing image-to-image transformation models such as the pix2pix model in neural decoding via the use of RFSimages. As such, this is the core difference between our work and the baseline methods. That is, this makes it possible for our method to exploit the underlying topographical structure in both stimuli and brain responses. Additional details of the experiments, additional results, and a link to the source code are provided in the Supplementary materials.

### 3.6. Performed experiments

This paper consists of seven experiments: (1) The brain2pix architecture trained on synthetic data with fixed receptive field locations, (2) training brain2pix using real fMRI data with a fixed receptive field for reconstruction, which we termed FixedRF, (3) training brain2pix on real data with learnable receptive fields for reconstruction, which we termed LearnedRF, (4) training traditional models for comparison with the brain2pix model (baseline experiment), (5) training brain2pix on fMRI data from various brain regions with fixedRF (ROI experiment), (6) removing essential components from the brain2pix architecture with fixedRF (ablation experiment), and (7) experimenting with various sizes of training data with a fixedRF.

Quantitative analysis comprised correlation values and distance values. These metrics were calculated by first passing each ground-truth and reconstructed frame through the pre-trained networks, Alexnet and C3D, to obtain the stimulus features and reconstruction features. Then the Pearson correlation coefficient and Euclidean distance were calculated between the ground-truth features and reconstruction features per frame. They were then averaged over all the frames in the test set.

Student's *t*-test was used to test if the mean reconstruction performance of the Brain2Pix model over the test set (*n* = 1,034) was significantly above chance level performance for each of the 12 evaluation metric combinations.

Binomial test was used to test if the overall reconstruction performance of the Brain2Pix model was significantly higher than a baseline model by taking every time any test set reconstruction metric (*n* = 1,034 × 12) of Brain2Pix model was higher than the baseline model as a “success” in the binomial distribution.

The code of implementation can be found in the Supplementary material in the GitHub repository ^1^.

## 4. Results

### 4.1. Brain2Pix on synthetic data

Before experimenting on real data, we used synthetic data to test feasibility and tune hyperparameters of brain2pix. Instead of mapping BOLD responses for each ROI and voxel onto the pixel space, we mapped target stimuli onto pixel space using the same exact method. This filtering of target images with the RF centers gave us the same amount of input pixels for the model, all at the same location. However, their activations were not based on actual brain signals, but rather on the pixels of the target images, which resulted in the same number of pixels as the number of voxels provided by the three brain regions used. We got clear reconstruction images from this, which confirmed that the number of RF pixels provided by V1 + V2 + V3 ROIs could theoretically carry enough spatial information for the model to generate realistic and accurate results.

### 4.2. Brain2Pix variants

The brain2pix variants described in detail below differed in how they transformed brain responses from volumetric representation to image representation.

#### 4.2.1. FixedRF: Providing the model with mapped out brain signals

**Figure 3:**
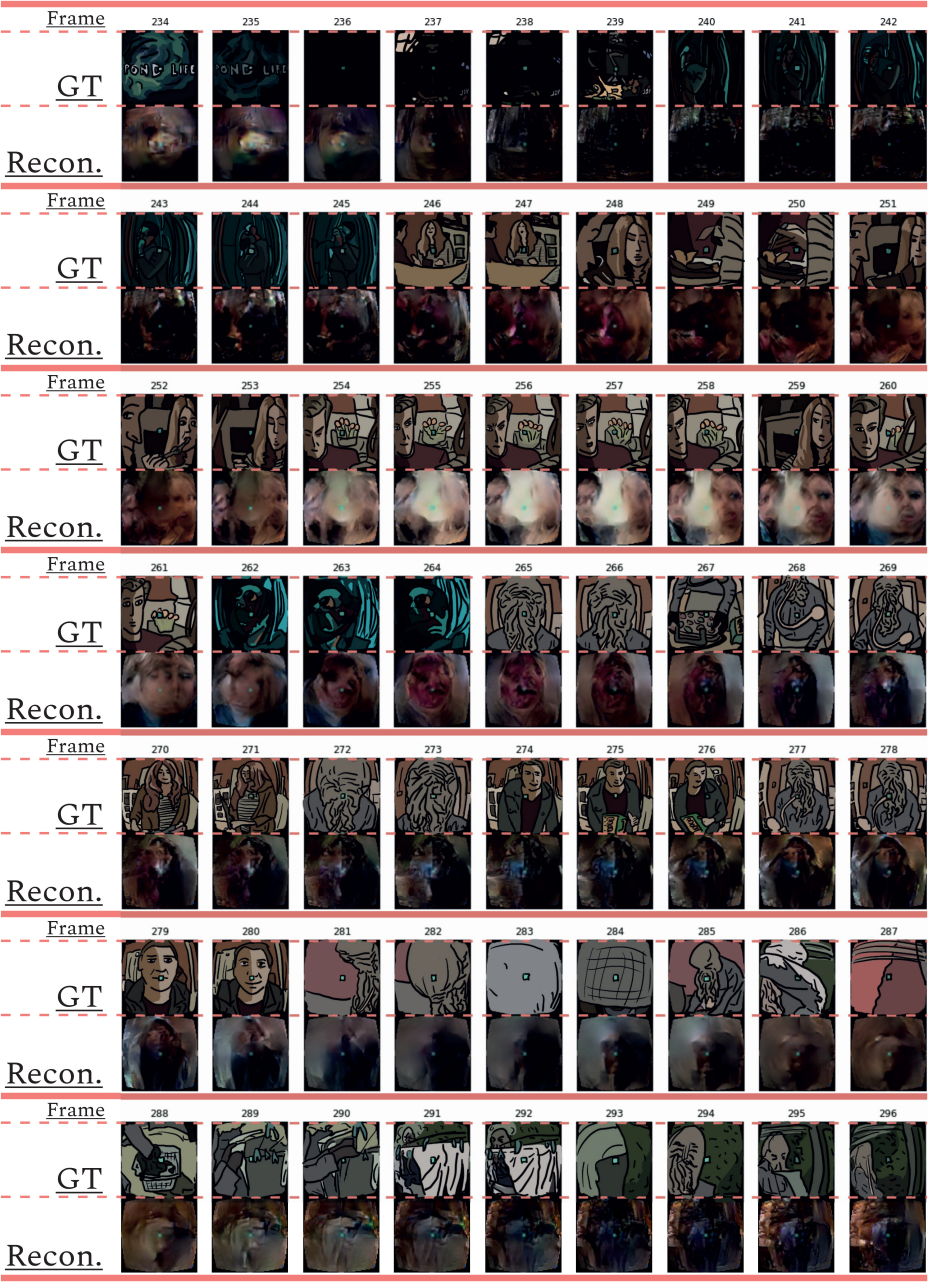
Sequential of reconstructed frames. Consecutive frame sequence from a video fragment of the test data [ground truth (GT)] and the corresponding reconstructions (Recon.).

**Figure 4:**
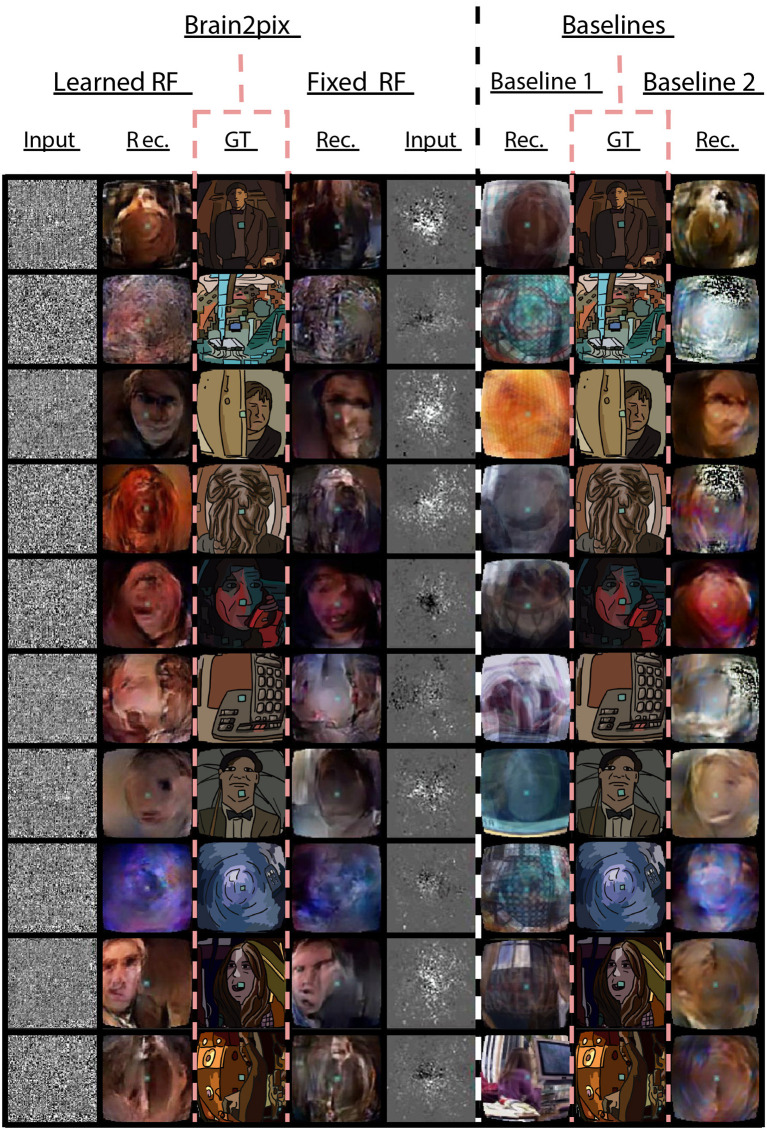
Baseline experiment. Comparison between the reconstructions of the brain2pix and the baseline models. The reconstructions of brain2pix are shown in columns 2 and 4, with their corresponding ground truth (GT) in the middle column 3, and inputs in columns 1 and 5. The reconstruction of the baseline models are shown in columns 6 and 8 together with their corresponding GT in the middle column 7.

The protocol for our main model is to use the (fixed) receptive field estimates. Once the brain signals were mapped onto visual space and the model was assembled, we ran it to obtain reconstructions (see Figures 3, 4 under Brain2pix > FixedRF). The results show individual frames from a snippet of the fMRI test set. The figure contains a selection of frames from the test set and their corresponding reconstructions. This figure shows that the model successfully reconstructed frames that contained head shapes, silhouettes, facial expressions, and also objects (such as a white blanket in frames 289–292). The figure also depicts a smooth transitioning between frames, which allows better reconstruction of video clips. Tables 1, 2 contain the quantitative results of the model under “B2P-FixedRF.” This model achieves the highest performance in terms of correlation for most of the studied feature layers. However, for the distance values, it shows the best results for only one layer.

**Table 1:** Baseline experiment *correlation values*.

	**B2P-learnRF**	**B2P-fixedRF**	**Baseline 1**	**Baseline 2**	**Baseline 3**
AlexNet pool2	0.4573	**0.4615**	0.2526	0.4141	0.0479
AlexNet pool5	**0.3555**	0.3507	0.2318	0.3269	-0.0394
AlexNet fc6	**0.4653**	0.4608	0.2016	0.4196	-0.0214
C3D pool2	0.4831	**0.4869**	0.3662	0.4212	0.1284
C3D pool5	0.2356	**0.2426**	0.0489	0.2030	0.0427
C3D fc6	0.2426	**0.2519**	0.0412	0.2185	0.0434

Correlation values between the features of the reconstructions of the baseline experiment and features of the target taken from various layers obtained from passing target/reconstructions through a pre-trained network (Alexnet and C3D). Here, comparisons are done for the brain2pix models and baseline models. Bold values indicate the highest correlation values.

**Table 2:** Baseline experiment *distance values*.

	**B2P-learnRF**	**B2P-fixedRF**	**Baseline 1**	**Baseline 2**	**Baseline 3**
AlexNet pool2	**4.6089**	4.6252	5.5266	5.3511	56.8820
AlexNet pool5	**1.2425**	1.2692	1.2432	1.2532	3.3593
AlexNet fc6	1.4062	**0.0899**	1.7322	1.4861	5.5175
C3D pool2	**1.3842**	1.3983	1.4238	1.8132	31.6262
C3D pool5	3.4850	3.4626	**2.6601**	3.8881	9.6941
C3D fc6	0.9269	0.9332	**0.8655**	1.1847	4.8835

Distances between reconstruction and target features of various layers obtained from passing reconstructions/targets through a pre-trained network (Alexnet and C3D). Here, comparisons are done for the brain2pix models and baseline models. Bold values indicate the lowest distance values.

#### 4.2.2. LearnedRF: Layers that output 2D acting as RFSimages

The second variant (referred to as LearnedRF) used a dense layer to perturb the image representation in the FixedRF variant as a function of the volumetric representation in order to improve it even further. Reconstructions of this model are shown in Figure 4 under Brain2pix > LearnedRF. We found that the LearnedRF variant has the highest performance in terms of correlation for the Alexnet pool2 and Alexnet fc6 layers (see Tables 1, 2).

The differences in the quantitative and qualitative results between LearnedRF and FixedRF were not large, suggesting that both the RF models capture the correct topographical structures. Both brain2pix variants contain significantly above chance level performance (*p* < 0.05; Student's *t*-test) and significantly outperformed both baselines that are described in the following sections (*p* < 0.05; binomial test) (Figure 4). By design, fc6 of Alexnet is very invariant and almost not retinotopic so learnable RF is likely introducing unnecessary complexity, which could be leading to overfitting in this one instance.

### 4.3. Baselines

#### 4.3.1. Baseline 1: Nishimoto-like model

The reconstructions (Rec.) resulting from this method are shown in Figure 4 in the columns under the header “Baselines > Baseline 1 > Rec.”. Unlike our method, this method did not make use of retinotopic information, and could be overfitted to the training image distribution. This resulted in difficulty of the model in reconstructing things that are not present in the training set, such as the Ood character. Additionally, some reconstructions do not come close to the target perceptually, such as the 10th row in Figure 4 where a computer is reconstructed although it is supposed to be a person. Quantitative results are shown in Tables 1, 2, which indicates all lower correlation values and mainly higher distances (except for C3D pool5 and C3D fc6) compared to our model. The correlations (performance estimation) are generally lower than Baseline 2, but higher than a model with only FC-layers, explained in the next subsection.

#### 4.3.2. Baseline 2: Shen-like model

The second baseline model we trained with an adversarial loss and a feature loss, which is based on the end-to-end reconstruction model from Shen et al. (2019a). We used the generator and discriminator modules present in brain2pix, however, we did not construct RFSimages for the input of the model. Instead, this baseline model takes as input the fMRI voxels that are z-scored and temporally aligned with the stimuli (same pre-processing steps as the original brain2pix), and the first layer is a linear layer as it does does not take in voxels that are mapped onto 2D space.

Reconstructions of this method is shown in Figure 4 below “Baselines > Baseline 2 > Rec.”. Although we trained both the models with the same amount of epochs and the same amount of data, quantitative and qualitative results show that our model outperforms the Shen et. al. baseline model (see Tables 1, 2). For instance, some static black and white noise is present in the reconstructions of row 2, 4, 6. Additionally, the Ood character is not recognizable. This model appeared to perform better than the Baseline 1 on the Doctor Who dataset. Our analyses indicates that our method outperforms both baselines.

#### 4.3.3. Baseline 3: Fully connected layers

We added another baseline where we replaced all of the convolutional layers of brain2pix with fully connected layers. The output of this model appear to be random noise and did not suggest that it would be capable of reconstructing images. Samples of the reconstructions of this baseline were not included since since they look like random noise and therefore were not informative.

### 4.4. ROI experiments: V1, V2, V3, V1–3

**Figure 5:**
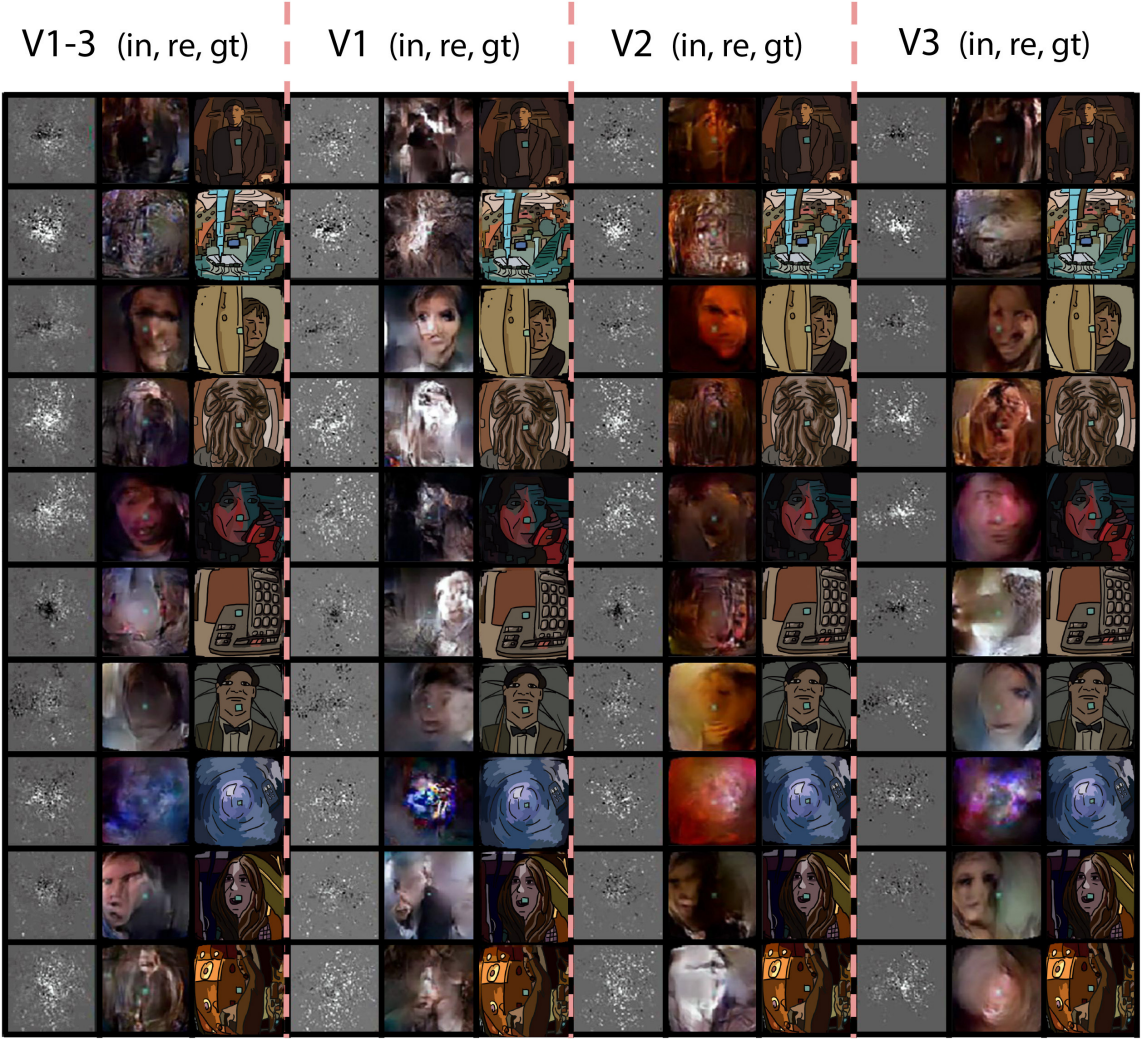
ROI experiment. Reconstructions from the brain2pix FixedRF method trained on various brain regions. Columns 1–3 show the inputs (in), reconstructions (re), and ground truths (gt) of all combined regions (V1–V3), respectively. Columns 4–6 show these results for only V1, columns 7–9 for V2, and finally columns 10–12 are results belonging to *t* he model trained only on V3.

In order to isolate the role of the regions of interest, we performed a series of follow up experiments where only data from one ROI was provided to the network. We used a fixed receptive field matrix (Equation 1). All the experimental details are identical to the main experiment. Figure 5 shows the ROI-specific reconstructions. Reconstructions based on V1 tend to have sharper pixelwise correspondence, whereas some high-level features, such as global illumination, were not captured very well. The combined brain2pix model with all ROIs (V1–V3) on the other hand was able to capture the color profile of the scenes very well. These two models generated images that captured further readily interpretable high-level information such as the existence of a person in the scene and even the expressions on the faces of individuals in the scenes. It is interesting to note that the ROIs did not contain higher-level brain regions, such as lateral occipital cortex that play a large role in object perception and fusiform face area that specializes in face processing.

The quantitative results are given in Tables 3, 4. The combined model performs substantially better than the individual models with the V1 model having the worst performance. V2 and V3 were similar to each other in quantitative performance. The poor performance of the model based on V1 can be due to two main reasons: Either (1) the adversarial and feature losses have larger weights in the training processes, biasing the model toward using higher-order features for reconstructions, or (2) V1 being less informative than downstream areas as far as the loss function is concerned.

**Table 3:** ROI experiment *correlation values*.

	**V1–3**	**V1**	**V2**	**V3**
AlexNet pool2	**0.4615**	0.2626	0.4247	0.4178
AlexNet pool5	**0.3507**	0.1920	0.3387	0.3294
AlexNet fc6	**0.4608**	0.2704	0.4453	0.4304
C3D pool2	**0.4868**	0.2640	0.4704	0.4685
C3D pool5	**0.2426**	0.1299	0.2224	0.2157
C3D fc6	**0.2519**	0.1349	0.2302	0.2228

Correlation between Alexnet and C3D features for the ROI experiment. V1, V2, V3 are the individual regions of interests and V1–V3 are all three visual layers combined. Bold values indicate the lowest/highest entries.

**Table 4:** ROI experiment *distance values*.

	**V1–3**	**V1**	**V2**	**V3**
AlexNet pool2	**4.6252**	5.9592	4.8401	5.1095
AlexNet pool5	1.2691	1.6985	**1.2673**	1.2898
AlexNet fc6	**0.0899**	2.0553	1.4820	1.4877
C3D pool2	1.3983	1.8996	**1.3949**	1.4288
C3D pool5	**3.4626**	4.5135	3.5756	3.5542
C3D fc6	**0.9332**	1.1592	0.9873	0.9417

Distances between Alexnet and C3D features for the ROI experiment. V1, V2, V3 are the individual regions of interests (ROIs) and V1–V3 are all three ROIs combined.

Bold values indicate the lowest/highest entries.

### 4.5. Ablation experiments: Removal of components from the model

The ablation studies were performed to test the impact of VGG-loss and adversarial loss on the performance of the model (see Tables 5, 6). “No adversarial” refers to the brain2pix without a discriminator loss, using only the VGG-feature loss to optimize the model. In this ablation case, the model did not learn to reconstruct images but rather outputted square patterns that repeated across all images. The second model is the “no feature” model, which was trained without the VGG-loss, thus only making use of the adversarial loss. This resulted in images that look like reconstructions but did not approximate the target.

**Table 5:** Ablation experiment *correlations values*.

	**brain2pix**	**No feature**	**No adversarial**
AlexNet pool2	**0.4615**	0.3950	0.1626
AlexNet pool5	**0.3507**	0.3168	0.1301
AlexNet fc6	**0.4608**	0.4273	0.1567
C3D pool2	**0.4868**	0.4396	0.16286
C3D pool5	**0.2426**	0.2123	0.0396
C3D fc6	**0.2519**	0.2255	0.0495

Correlation between Alexnet and C3D features of the test stimuli and their reconstructions. The brain2pix is compared with two models with either no feature loss or no adversarial loss. Bold values indicate the lowest/highest entries.

**Table 6:** Ablation experiment *distance values*.

	**brain2pix**	**No feature**	**No adversarial**
AlexNet pool2	**4.6252**	5.6450	13.1021
AlexNet pool5	**1.2691**	1.4201	1.5935
AlexNet fc6	**0.0899**	1.5991	2.1505
C3D pool2	**1.3983**	1.5988	7.5023
C3D pool5	**3.4626**	3.6445	5.6622
C3D fc6	**0.9332**	0.9657	2.7328

Distances between reconstruction and target features of various layers obtained from passing through a pre-trained network (Alexnet and C3D). Here, comparisons are done for the brain2pix models and ablation models.

Bold values indicate the lowest/highest entries.

### 4.6. Experiment with various data sizes

**Figure 6:**
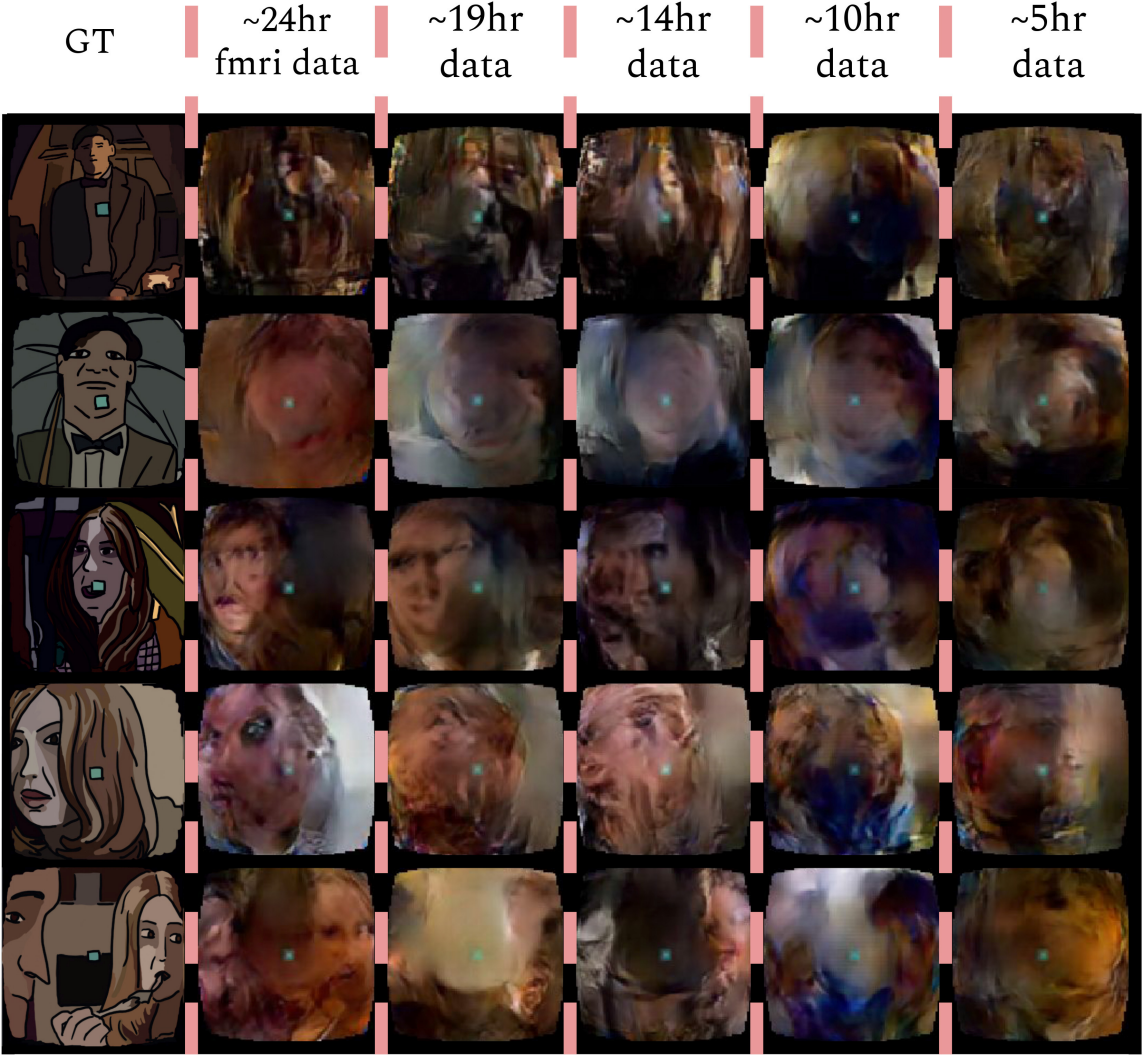
Time experiment. Reconstructions from the brain2pix fixedRF method trained on various amounts of fMRI data after 50 epochs of training. Ground truth (GT), ~24 h is the entire dataset, ~19 h is $\frac{4}{5}$, ~14 h is $\frac{3}{5}$, ~10 h is $\frac{2}{5}$, ~5 h is $\frac{1}{5}$ of the dataset.

Finally, we wanted to see how the model performs when less data is fed into the network. This was done by training our model on a selected amount of Doctor Who frames from the training set. The full training set, which was worth ≈24 h of data was split into the following: $\frac{4}{5}\approx 19h,\;\frac{3}{5}\approx 14h,\;\frac{2}{5}\approx 10h,\;\frac{1}{5}\approx 5h\;$ which resulted in training sizes of 95.200, 71.400, 37.600, and 23.800 frames with 50 epochs for each condition. The reconstructions of the splitted data is then compared with the full ~ 24 h worth of data (also trained on 50 epochs). The reconstructions of this experiment are shown in Figure 6. Although the highest amount of data shows the best reconstructions, we still see notable reconstructions based on smaller amounts of data, which suggests that it is not necessary to record 23 h worth of data to achieve reconstructions with the brain2pix model. The quantitative results can be found in Tables 7, 8.

**Table 7:** Splitted data *correlation* values: the model was trained on data worth of 5, 10, 14, 19, and 24 h.

	**B2P (~24 h**	**B2P (~19 h)**	**B2P (~14 h)**	**B2P (~10 h)**	**B2P (~5 h)**
AlexNet pool2	**0.4615**	0.4388	0.4262	0.4132	0.4342
AlexNet pool5	**0.3507**	0.3289	0.3236	0.3195	0.3206
AlexNet fc6	**0.4608**	0.4399	0.4270	0.4339	0.4376
C3D pool2	**0.4869**	0.4797	0.4270	0.4558	0.4714
C3D pool5	**0.2426**	0.2037	0.1933	0.2061	0.2057
C3D fc6	**0.2519**	0.2171	0.2061	0.2325	0.2226

Bold values indicate the lowest/highest entries.

**Table 8:** Splitted data *distance* values: the model was trained on data worth of 5, 10, 14, 19, and 24 h.

	**B2P (~24 h)**	**B2P (~19 h)**	**B2P (~14 h)**	**B2P (~10 h)**	**B2P (~5 h)**
AlexNet pool2	4.6252	4.5781	4.6371	4.8827	**4.4931**
AlexNet pool5	1.2692	1.2459	1.2602	**1.2399**	1.2485
AlexNet fc6	**0.0899**	1.4191	1.5028	1.4302	1.4385
C3D pool2	1.3983	1.3372	1.3657	1.4069	**1.3307**
C3D pool5	**3.4626**	3.6748	3.6790	3.6987	3.6451
C3D fc6	**0.9332**	0.9942	1.0039	1.0900	1.0114

Bold values indicate the lowest/highest entries.

## 5. Discussion

In this paper, we introduced a new neural decoding method for reconstructing video frames, which we call brain2pix, exploiting the receptive field mapping of visual areas by mapping brain activation to a linear pixel space where it is then processed with a fully convolutional image-to-image network. To the best of our knowledge, this is the first end-to-end approach capable of generating semantically accurate reconstructions from a naturalistic continuous video stream. Furthermore, our approach was shown to outperform other baseline decoding methods.

Since our method was applied on fMRI data, it also inherits the modality specific limitations common to all methods for visual decoding in fMRI.

First, the BOLD signal is an indirect measurement of the population receptive field activity rather than a direct single neuron measurement. As such, the stimulus-feature-response mapping embodied by the model might have modality dependent discrepancies compared to the true mapping defined by the underlying neuronal activity.

Second, the relatively low signal-to-noise ratio of visual stimulus driven BOLD signal dictates certain experimental decisions that can be considered not ideal. As such, the fMRI data in the test set were averaged over their 22–26 repetitions in contrast to the single trial fMRI data in the training set, which is a very common procedure in the neural coding literature (Kay et al., 2008; Nishimoto et al., 2011; Güçlütürk et al., 2017; Horikawa and Kamitani, 2017a; Dado et al., 2020). The rationale behind this procedure is to be able to sample as much of the stimulus space as possible in the training set for generalizability and increase the signal-to-noise ratio as much as possible in the test set for statistical power. Consequently, this allows the model to cover a wider spectrum of the stimulus–response relationship by learning the underlying features and still be evaluated with sufficient statistical power on a subset thereof.

Similarly, the scarcity of certain stimulus features in the training set typically causes a bottleneck in neural coding, which prevents accurate estimation of stimulus response mappings. For example, it has been previously shown in an encoding setting that the accuracy of predicting voxel responses from DNN features is positively correlated with the mean activity of those DNN features across the training set (Güçlü and van Gerven, 2015). We can also observe manifestations of this phenomenon in present work. For example, qualitative analysis shows that our model cannot accurately reconstruct certain objects like text. The most likely explanation of this result is the lack of sufficient frames with text or text features in the training set which should be expected considering the nature of the stimulus material.

Moreover, while our model performs better than baselines in general, it too suffers from some failure modes that can be qualitatively seen in the reconstructions. For example, our model tends to reconstruct some less frequent objects as faces, likely because faces are one of the most frequent in the training set.

Finally, even though the stimulus material has big differences from episode to episode, it still shares many commonalities between training and test sets. A more stringent evaluation strategy could be to have a test set sampled from a completely different material.

One of the next challenges that we can try to tackle is decoding at higher frame rates rather than using the same number of frames as brain signals in order to reconstruct even finer temporal details. Additionally, in our current experiments we focused mainly on optimizing our model based on responses from the early visual regions V1, V2, V3. A natural extension of the current work is to extend our focus to the higher level areas in the temporal and parietal cortex. Since these areas process coarse-grained semantic information, experiments feeding their responses to deeper layers of the network could reveal reconstructions driven by semantics. We can also apply the model on imagery data.

Neural decoding studies are crucial for understanding the functioning of the human brain, broadly benefiting the field of neuroscience. Furthermore, neural decoding algorithms make up a major component of brain-computer interfaces (BCIs). Brain-computer interfaces enable disabled people to perform tasks that they would not be able to perform otherwise, by substituting their lost faculties. These technologies can range from a communication interface for a locked-in patient, to a neuroprosthetic limb, and more. While the algorithms that we develop and study in this paper are specialized to reconstruct visual stimuli from brain responses, we foresee that the suggested principles can be applied to different applications, with some adaptations. For instance, we use a relatively slow signal (BOLD response), which reflects the neural responses that take place several seconds prior to them. A time-critical BCI system would need to make use of a signal with no such delays to perform well.

While admittedly the promise of algorithms that reconstruct internally generated or externally induced percepts is yet to be fully achieved, scientists that attempt to extract information from the brain should ensure the safety and privacy of the users (Ienca et al., 2018). Future studies should make sure to follow similar strict regulations, ensuring only a positive impact of these fascinating methods that allow us to peek into the human mind.

## Data availability statement

The original contributions presented in the study are included in the article/Supplementary material, further inquiries can be directed to the corresponding author/s.

## Ethics statement

The studies involving human participants were reviewed and approved by CMO regio Arnhem-Nijmegen, The Netherlands, CMO code 2014-288 with amendment NL45659.091.14. The patients/participants provided their written informed consent to participate in this study.

## Author contributions

UG, MG, YG, and LA designed the experiment. UG and KS collected the data. LL analyzed the data and prepared the figures. UG and LL wrote the main manuscript text. YG created the representative artwork in the figures. All authors reviewed the manuscript.

## Conflict of interest

The authors declare that the research was conducted in the absence of any commercial or financial relationships that could be construed as a potential conflict of interest.

## Publisher's note

All claims expressed in this article are solely those of the authors and do not necessarily represent those of their affiliated organizations, or those of the publisher, the editors and the reviewers. Any product that may be evaluated in this article, or claim that may be made by its manufacturer, is not guaranteed or endorsed by the publisher.

## Footnotes

^1^ https://github.com/neuralcodinglab/brain2pix
