# Supplementary material for: Brain2Pix: Fully convolutional naturalistic video frame reconstruction from brain activity
Source: Front Neurosci. 2022 Nov 14;16:940972. doi: 10.3389/fnins.2022.940972 (PMC9703977; doi:10.3389/fnins.2022.940972)
Supplement: Supplementary file 1 [file Data_Sheet_1.pdf]

Supplementary Material

1 INTRODUCTION

In this supplementary document, we provide additional results of analysis that were performed on the various baseline models. First, correlations between frames and reconstructions were made using the features of the pool2, pool5 and fc6 layers of the alexnet and c3d models. The first section contains figures. In subsection 2.1 these correlations were plotted and visualized. We have also ran the brain2pix model with undistorted stimuli and added the undistorted and distorted video frames and reconstructions in subsection 2.2. Finally, reconstructions from the ablation experiment are included in 2.3 of this document, which methods are described in section 4.5 in the manuscript.

The second section shows tables. Subsection 3.1 shows the means of the diagonal calculated and compared with the mean values that lie outside of the diagonal. This shows how well the reconstructions were correlated with its corresponding target for each model. Finally, in section 3.2, we ran additional comparisons that represent global illumination, color-profile and pixel-wise quantitative evaluations.

2 FIGURES

2.1 Visualization of the Pearson correlation coefficient between the reconstructions and targets of each frame

In the Figures S1, S2, S3 and S4, correlation matrices are shown between the features of reconstructions vs. the features of targets at all timepoints to indicate whether the reconstructions best represent it's target. On the axes of each plot, the correlation value between reconstructions (x-axis) and targets (y-axis) at timepoint $(t_0, ..., t_{1034})$ are shown starting from the top left of the plot.

The following plots were made by first passing each reconstruction and target frame into the pre-trained alexnet and c3d models to obtain the features from the pool 2, pool 5, and fc6 layers. Then each of these features were flattened and the Pearson correlation coefficients were calculated, using the sstats.pearsonr method in Python. The Pearson correlation coefficient indicates the linear relationship between two datasets. In the Figures S1, S2, and S4, a diagonal line is visible in the plot of each layer, which indicates that the reconstructions are representing its corresponding target to some degree. Based on the plots in Figure S3, Baseline 1 seems to not have well-represented reconstructions.

[Figure 1 about here.]

[Figure 2 about here.]

[Figure 3 about here.]

[Figure 4 about here.]

2.2 Non-distorted stimuli and reconstructions

The reconstructions of the paper are based on targets that underwent a retinawarp. In Figure S5 we show the targets that have not undergone a retnawarp. In Figure S6, we show the same targets but with the

retinawarp filter. Figures S7 and S8 show the reconstructions of the images based on non-distorted and distorted targets, relatively.

[Figure 5 about here.]

[Figure 6 about here.]

[Figure 7 about here.]

[Figure 8 about here.]

2.3 Reconstructions from the ablation experiment

These are the results from the ablasion experiment that is described in section 4.5 of the manuscript. In the figure we show 5 conditions. The first column called ”GT” shows the ground truth images (frames from the videos). The second column ”no-vgg” includes reconstructions of the brain2pix where the loss was adjusted to be only an adversarial loss. In the third column ”no-gan”, we show the reconstructions where there was no adversarial loss. We also ran the experiment without using a loss, which is shown in the fourth column ”no-loss”. And lastly, we show the reconstructions of the complete model in the last column ”b2p”.

[Figure 9 about here.]

3 TABLES

3.1 Linear relationships between reconstruction and target

The mean values of the diagonal are calculated using the plots from section 2.1 above. The values along the diagonal are expected to be the highest because those values indicate how well the reconstructions are corresponding to its target. If the diagonal is not higher than the non-diagonal, then the reconstructions can evenly correspond to any arbitrary frames in the dataset, which is not the objective of the model. We can see that in Tables S1, S2, S4, the diagonals are higher than the non-diagonals. Whereas in Table S3, the diagonals do not seem to be higher than the non-diagonals. This means that the brain2pix models and baseline 2 did reconstruct images that are representative of the stimuli, whereas baseline 1 did not.

[Table 1 about here.]

[Table 2 about here.]

[Table 3 about here.]

[Table 4 about here.]

3.2 Additional evaluations (global illumination, color-profile, pixel-wise)

Additional quantitative evaluations were conducted to show that that this method is capable of generating semantically accurate reconstructions. For quantifying the luminance we first converted the images to black and white and then obtained the correlation values and euclidian distances between the reconstructions and targets. For HSV, the targets and recosntructions were converted from RGB to HSV and values were

obtained in the same manner. Finally, for pixel-wise evaluations, there were no filters applied, values were calculated from the RGB targets and reconstructions. The resulting values are shown in Tables S5 and S6.

[Table 5 about here.]

[Table 6 about here.]

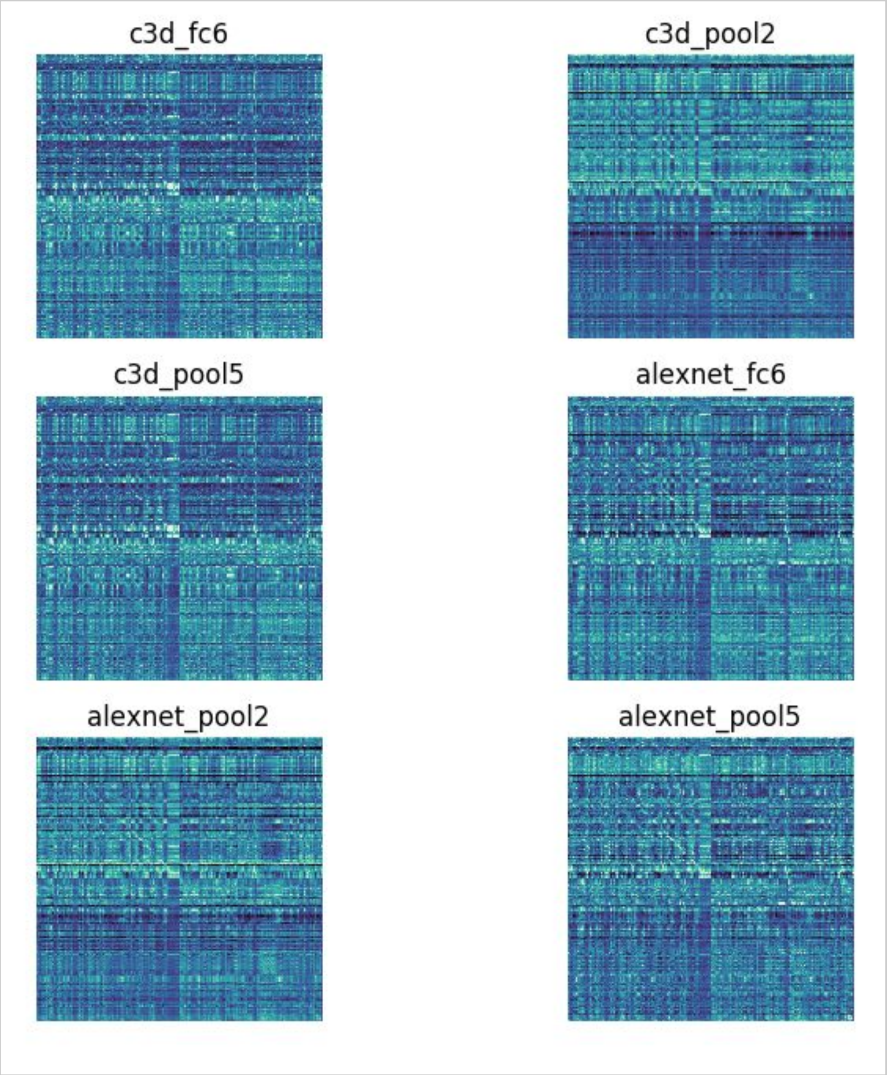

Figure S1: B2P Fixed RF

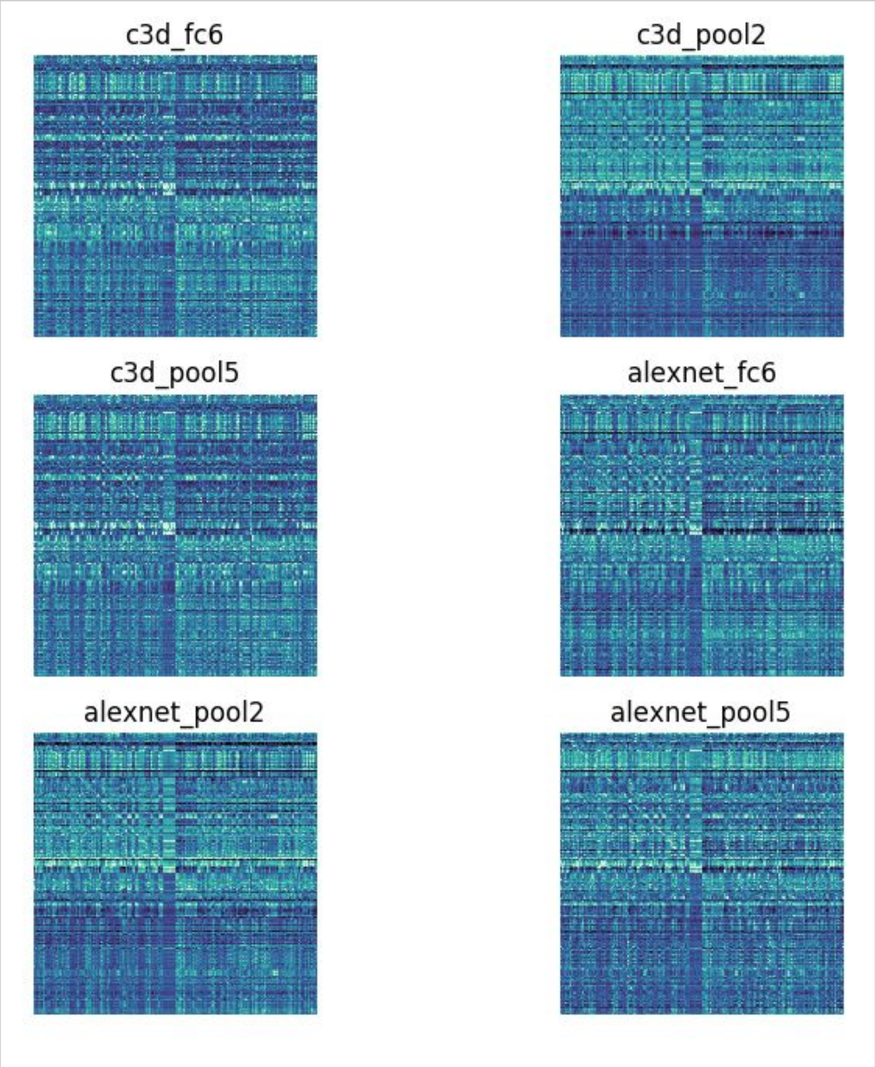

Figure S2: B2P Learned RF

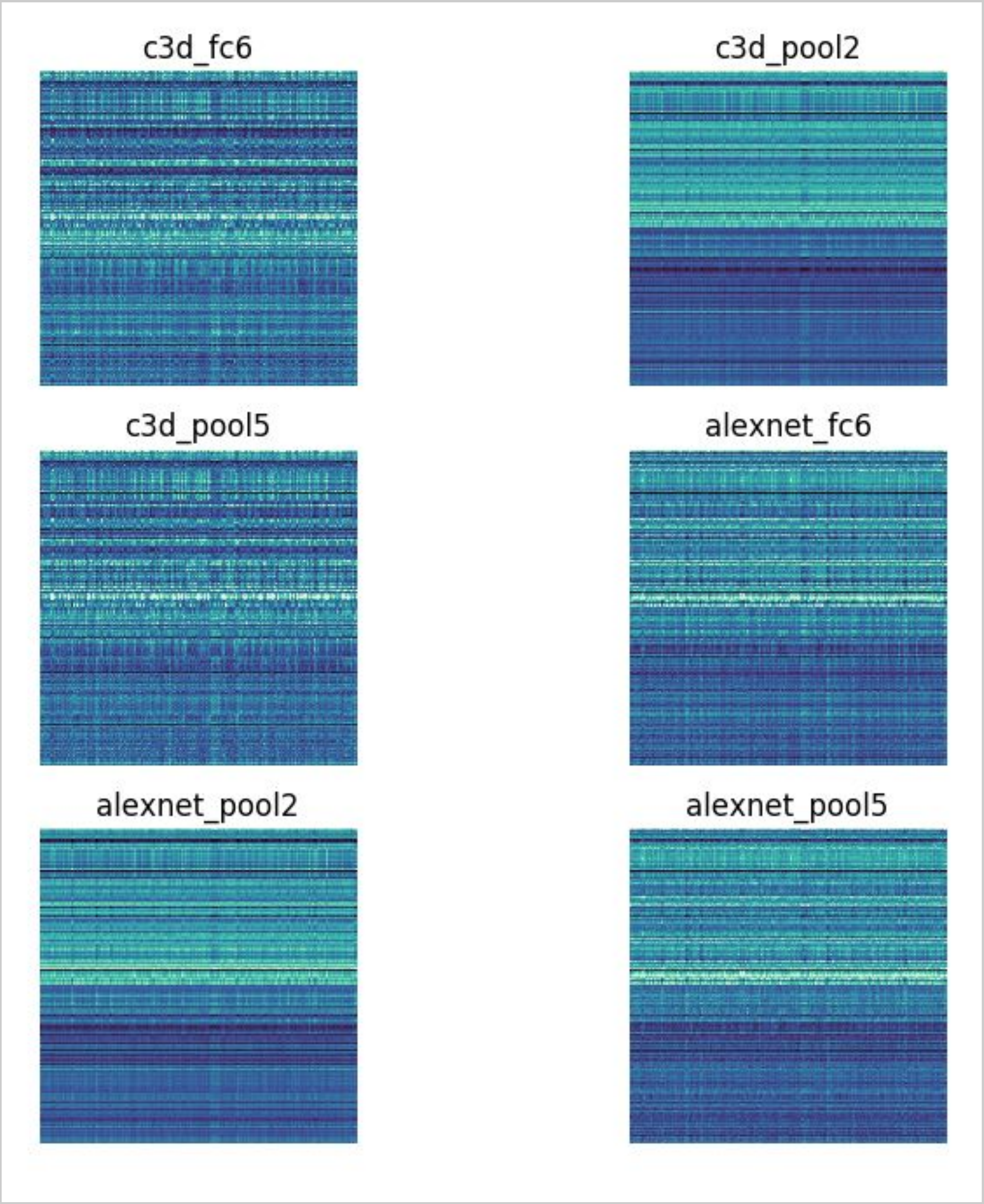

Figure S3: Baseline 1: Nishimoto-like model

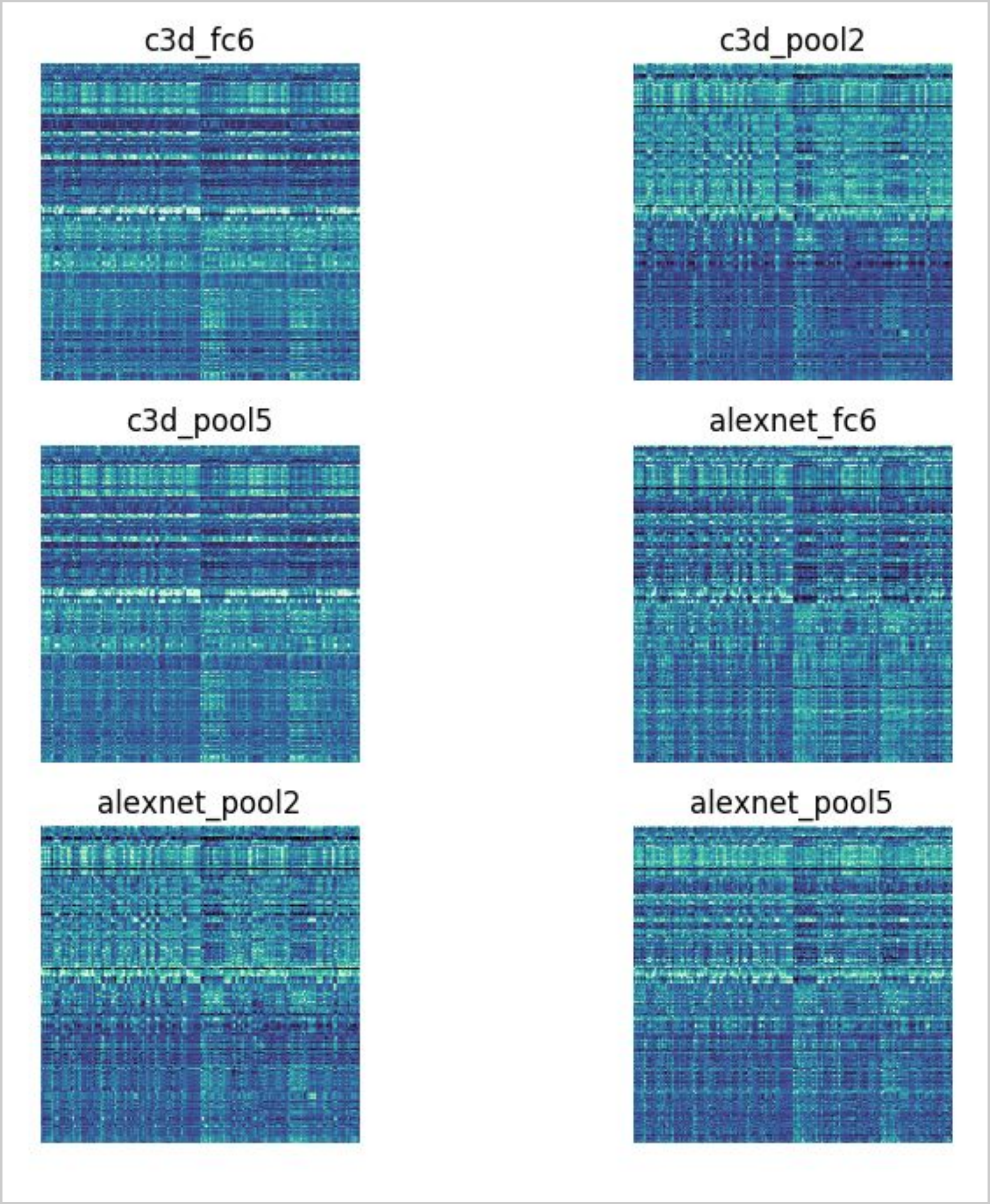

Figure S4: Baseline 2: Shen-like model

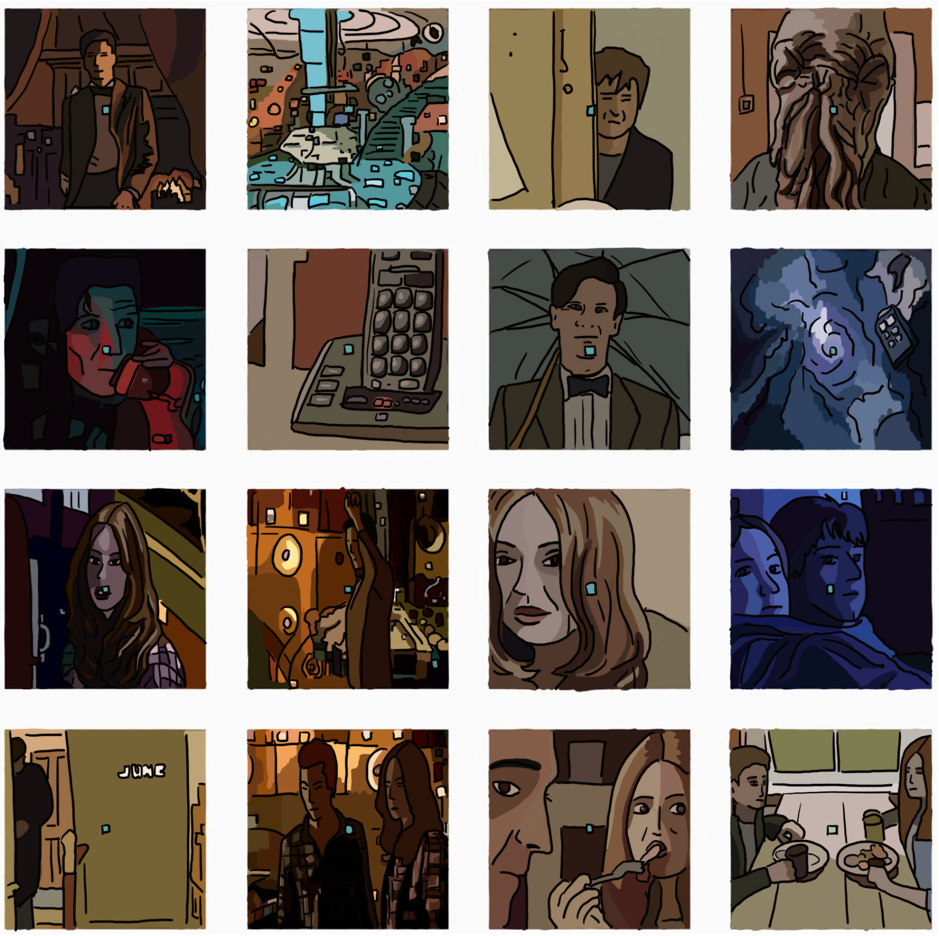

Figure S5: Non-distorted stimuli (drawing)

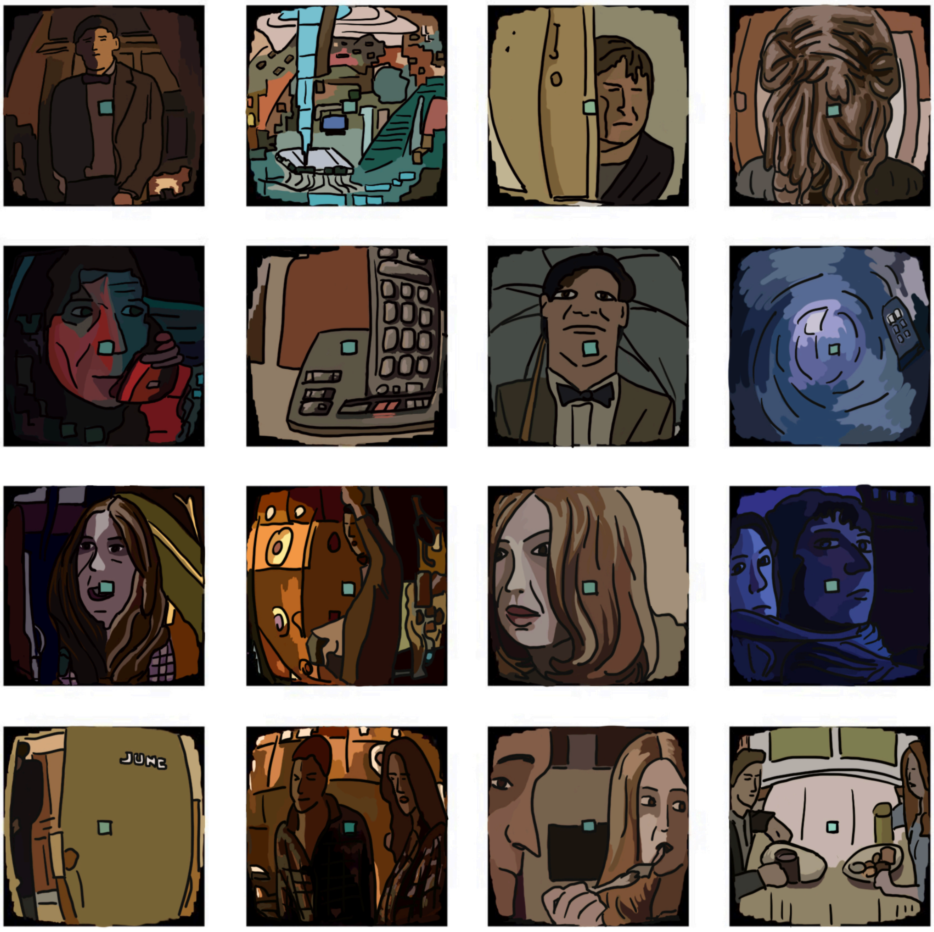

Figure S6: Distorted stimuli (drawing)

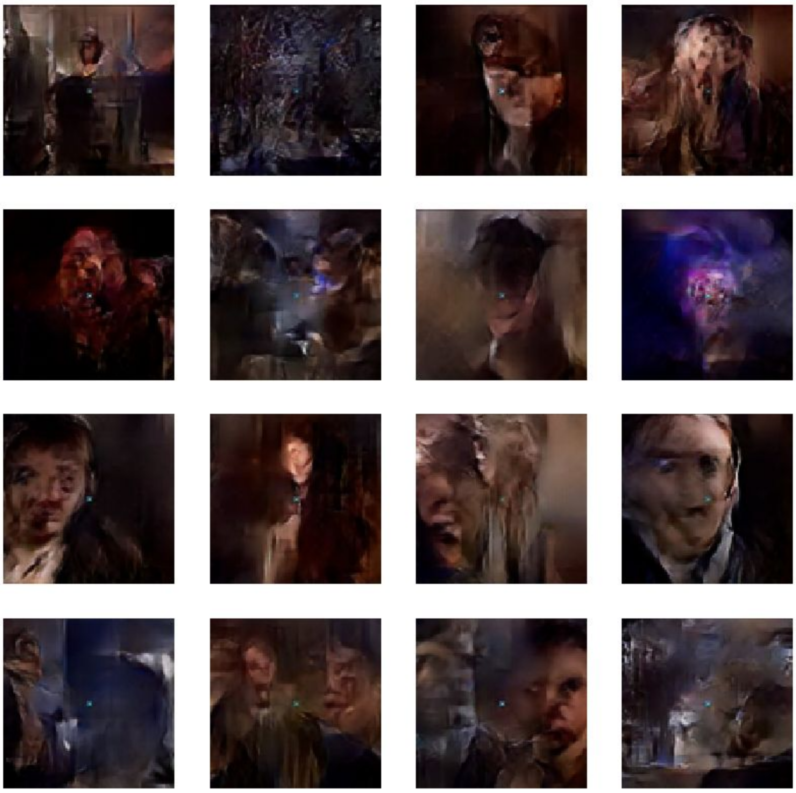

Figure S7: Reconstructions based on non-distorted stimuli

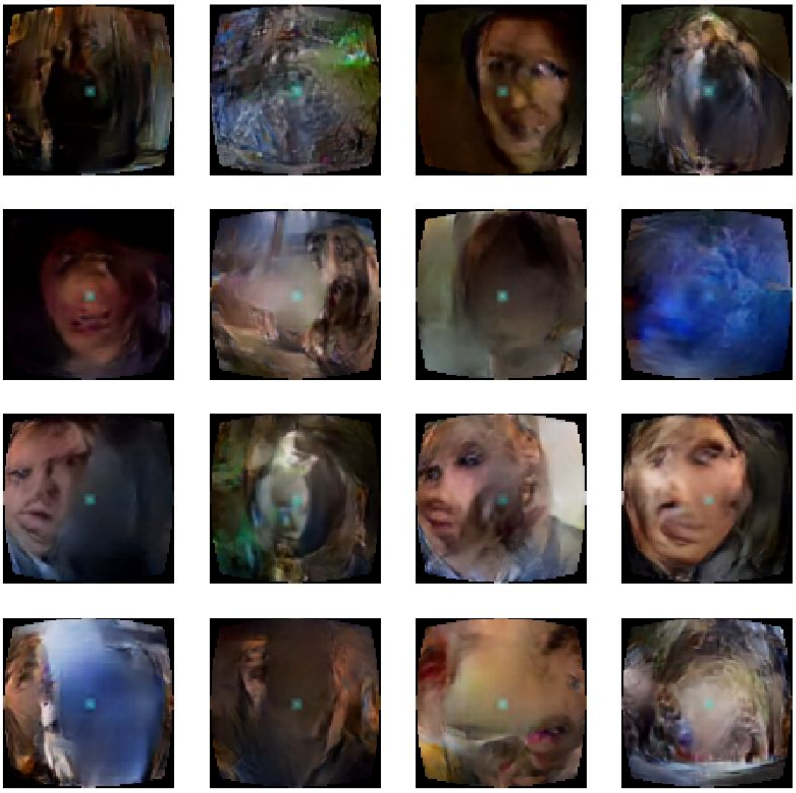

Figure S8: Reconstructions based on distorted stimuli

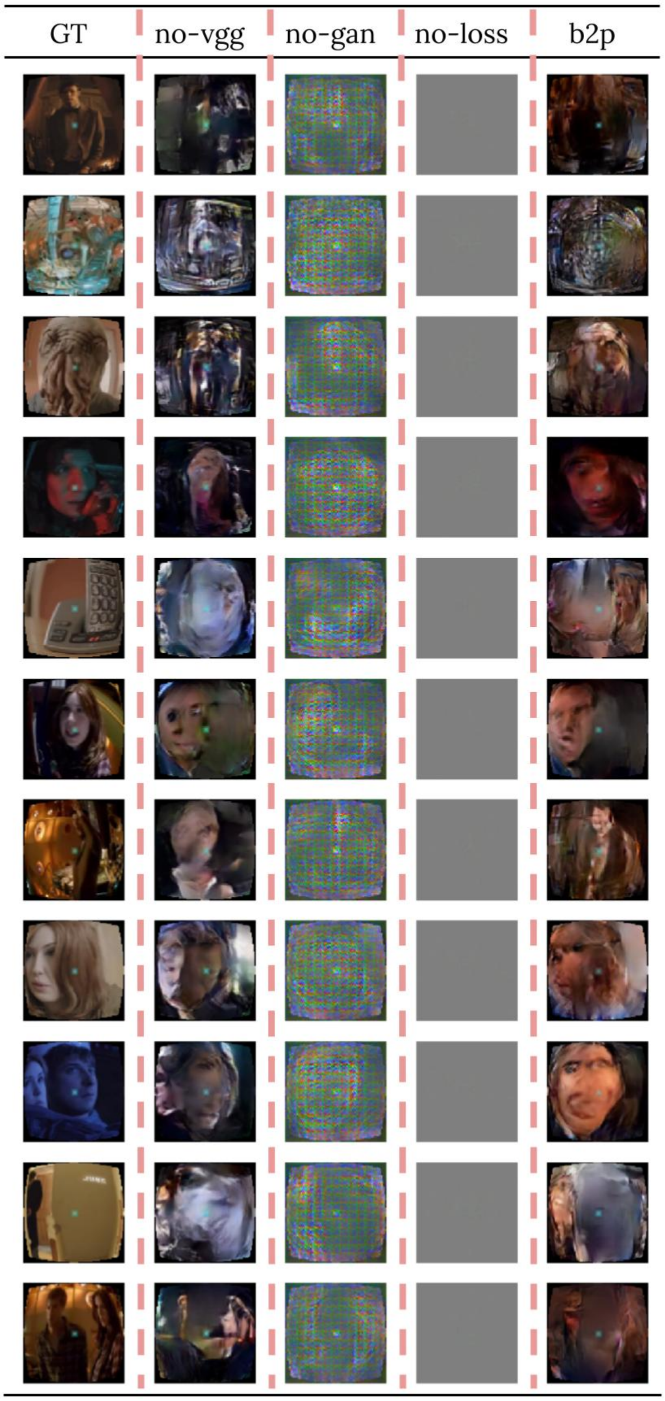

Figure S9: Ablation experiment

Table S1. B2P Fixed RF. Diagonal (D) vs Non-diagonal (ND).

	**Diagonal**	**Non-diagonal**
c3d_fc6	0.6476	-0.0024
c3d_pool2	0.6699	-0.0015
c3d_pool5	0.8122	-0.0008
alexnet_fc6	0.8058	-0.0008
alexnet_pool2	0.8877	-0.0009
alexnet_pool5	0.9836	-0.0010

Table S2. B2P Learned RF. Diagonal (D) vs Non-diagonal (ND).

	**D**	**ND**
c3d_fc6	0.6793	-0.0031
c3d_pool2	0.6922	-0.0020
c3d_pool5	0.8811	-0.0039
alexnet_fc6	0.8499	-0.0016
alexnet_pool2	0.9496	-0.0013
alexnet_pool5	0.9607	-0.0026

Table S3. Baseline 1. Diagonal (D) vs Non-diagonal (ND).

	**Diagonal**	**Non-diagonal**
c3d_fc6	-0.0102	-0.0019
c3d_pool2	0.0096	0
c3d_pool5	0.0036	-0.0040
alexnet_fc6	-0.0026	-0.0011
alexnet_pool2	0.0145	0.0002
alexnet_pool5	-0.0017	-0.0010

Table S4. Baseline 2. Diagonal (D) vs Non-diagonal (ND).

	**Diagonal**	**Non-diagonal**
c3d_fc6	0.4179	-0.0032
c3d_pool2	0.7556	-0.0021
c3d_pool5	0.5291	-0.0040
alexnet_fc6	0.6016	-0.0018
alexnet_pool2	0.9693	-0.0010
alexnet_pool5	0.6797	-0.0022

Table S5. Correlation values

	V1	***V2***	*V3*	*V1-3*	*B1*	*B2*	*learnedRF*
Luminance	0.2022	0.3322	0.3426	0.3927	0.3058	0.3899	**0.4176**
HSV	-0.0324	**0.3400**	0.2764	0.3054	0.1176	0.1512	0.2682
Pixel	0.1870	0.3373	0.3382	0.3890	0.3195	0.3715	**0.4061**

Table S6. Euclidian Distances

	V1	***V2***	*V3*	*V1-3*	*B1*	*B2*	*learnedRF*
Luminance	102.1224	**89.8638**	91.2339	90.1622	95.9528	91.7151	90.2375
HSV	103.3783	**89.5440**	90.7668	91.1263	93.0515	94.2848	90.8036
Pixel	102.3654	**89.8742**	91.1480	90.1312	96.2516	92.4507	90.4305
